# Hysteroscopic diagnosis and excision of myometrial cystic adenomyosis

**DOI:** 10.1007/s10397-014-0861-5

**Published:** 2014-10-03

**Authors:** S. Gordts, R. Campo, I. Brosens

**Affiliations:** Leuven Institute for Fertility & Embryology, Tiensevest 168, 3000 Leuven, Belgium

**Keywords:** Adenomyosis, Cystic, Hysteroscopy, Diagnosis, Treatment

## Abstract

In 1908, Cullen described the first cases of cystic adenomyosis in his textbook on adenomyomata. Although not very common, with the introduction of noninvasive imaging techniques such as magnetic resonance imaging (MRI) and 3-D transvaginal ultrasound, an increasing number of cases have been reported. Patients primarily complain of severe dysmenorrhea, chronic pelvic pain, and dysfunctional uterine bleeding. Currently, it is unclear whether adenomyosis and, more specifically, cystic adenomyosis can be an underlying reason for impaired fertility and reproductive outcome. With the postponement of childbearing, the number of patients with adenomyosis and cystic adenomyosis seeking fertility treatment is increasing. Therefore, in these patients, uterine exploration should include not only the evaluation of the endometrial cavity but also the exploration of the sub-endometrial zone. Indirect imaging techniques, combined with office mini-hysteroscopy, offer the possibility of complete uterine exploration. Two patients with cystic adenomyosis are described in this paper: one had the chief complaint of menorrhagia and the other was referred for evaluation of infertility and severe dysmenorrhea. The aim of these case reports is to present hysteroscopic dissection and ablation of adenomyotic cysts as an alternative procedure for the surgical management of this condition.

## Background

The uterine adenomyotic cyst is a cystic structure lined with endometrial tissue and surrounded by myometrial tissue that, in most cases, contains hemorrhagic material. Cullen [1] described the first cases in 1908 in his textbook on adenomyomata, in which he distinguished submucosal and subperitoneal cystic adenomyomata. In contrast to diffuse adenomyosis, the disease primarily occurs in adolescents and women younger than 30 years and is not associated with diffuse uterine adenomyosis [2].

The clinical symptoms are nonspecific and include dysmenorrhea starting at an early age around the time of menarche, chronic pelvic pain, and dysfunctional uterine bleeding. The dysmenorrhea tends to progressively increase and is resistant to therapy with analgesics or cyclic oral contraceptives. When viewed with ultrasound, the cyst increases in size at the time of menstruation and hormonal suppression with continuous oral contraceptive pills results in a partial regression [3].

Since the introduction of imaging techniques, an increasing number of cases have been described in adolescents and young adult women with untreatable dysmenorrhea. Currently, the diagnosis is primarily based on magnetic resonance imaging (MRI) criteria or 3-D ultrasound; it appears as a cystic structure with an internal diameter of ≥10 mm, with hemorrhagic content surrounded by myometrial tissue. The disease has received significant attention in Japan where more than 11 cases of cystic adenomyomata have been described [4, 5].

In a review of cystic adenomyosis of the uterus, Brosens et al. [2] defined three subtypes (A, B, and C) according to the location of the cyst and complexity of the lesion: subtype A1 includes the submucous or intramural cystic adenoma, subtype A2 includes cases with a cystic polypoid lesion, subtype B1 includes subserous cystic adenomyosis, subtype B2 includes cases with exophytic growth, and subtype C comprises uterine-like masses.

Medical treatment in adolescents includes the continuous use of an oral contraceptive [2, 4]. The use of a progestogen-loaded intrauterine device has been suggested for subtype A cystic adenomyosis [6]. In young women under menstruation-suppression therapy, the cyst has been shown to regress [3].

In most cases, surgical excision is performed via laparotomy; however, in recent years, laparoscopy has been employed [4, 5]. Hysteroscopy is commonly used for exploring the uterine cavity [7–9]. The cyst may bulge into the uterine cavity, and changes, such as abnormal vascularization or fibrosis, may be observed in the endometrium at the site of the cyst. Lowering the intrauterine pressure is helpful for a better identification of the submucosal cystic structures.

Ryo et al. [10] used a radio-frequency needle inserted via the cervix under ultrasound guidance for ablation of the cyst. Giana et al. [11] described hysteroscopic removal of a polypoid adenomyotic cyst. The 2-cm cystic mass arising from the posterior right lateral wall of the uterus was surrounded by hemorrhagic endometrium and a thin layer of myometrium. The cyst did not appear to communicate with the endometrial cavity, and it was deflated by aspiration of a brownish yellow dense fluid. The cyst wall was removed. Microscopic examination revealed that the cyst was lined by endometrial epithelium and several adenomyotic foci. The aim of these two case reports is to highlight the possible use of operative hysteroscopy, using mechanical dissection and ablative bipolar current, as an alternative surgical option for the treatment of cystic adenomyosis.

## Material and methods

Mini-hysteroscopes are commonly used for the exploration of the uterine cavity. Through a gliding system, the Trophy° hysteroscope (Karl Storz, Germany) offers the possibility to change from a diagnostic 2.9-mm hysteroscope to an operative 4.4-mm scope without the need to remove the hysteroscope. Through the operative channel, 5-Fr instruments (scissors, bipolar needle, and bipolar coagulation probes) are used for dissection or coagulation.

**Fig. 1:**
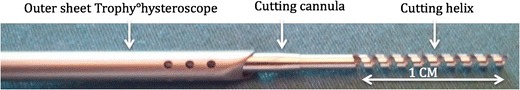
Utero-Spirotome mounted in outer sheet of Trophy° hysteroscope

In addition to the direct visualization of the uterine cavity, the hysteroscopic approach offers the possibility of obtaining endometrial/myometrial biopsies under visual control and/or ultrasound guidance. The biopsy of focal endometriotic lesions and/or a hyperplastic junctional zone is technically difficult, if not impossible, to perform. A new device, the Utero-Spirotome (Fig. 1), offers the possibility of a direct and frontal (D&F) tissue harvest. This procedure allows the biopsy of endomyometrial layers. Significant experience with this biopsy system exists in breast applications where efficacy and safety have been fully documented (12, 13). The Utero-Spirotome operates with two devices in tandem: the receiving needle with a cutting helix at the distal end, and a cutting cannula as an outer sheet. The correct direction and position of the helix point is under continuous ultrasonographic imaging and hysteroscopic control. Under ultrasound guidance, the spirotome can be directed towards any intramural localized lesion such as cystic adenomyosis, thus creating a visible hysteroscopic channel that allows access to the cystic cavity.

Both patients gave their written informed consent.

### Case 1

A 44-year-old woman was referred to our unit with a history of secondary infertility of 12-month duration. Hysteroscopy revealed the presence of an intracavitary 2-cm myoma, which was removed at that time by an operative hysteroscopic procedure. After the second IVF cycle, the patient became pregnant; however, the pregnancy was terminated as a missed abortion at 8-week gestation. Because of her age, she had no further desire for pregnancy. In 2007, the patient, now 51 years old, was again referred to us for evaluation of menorrhagia.

**Fig. 2:**
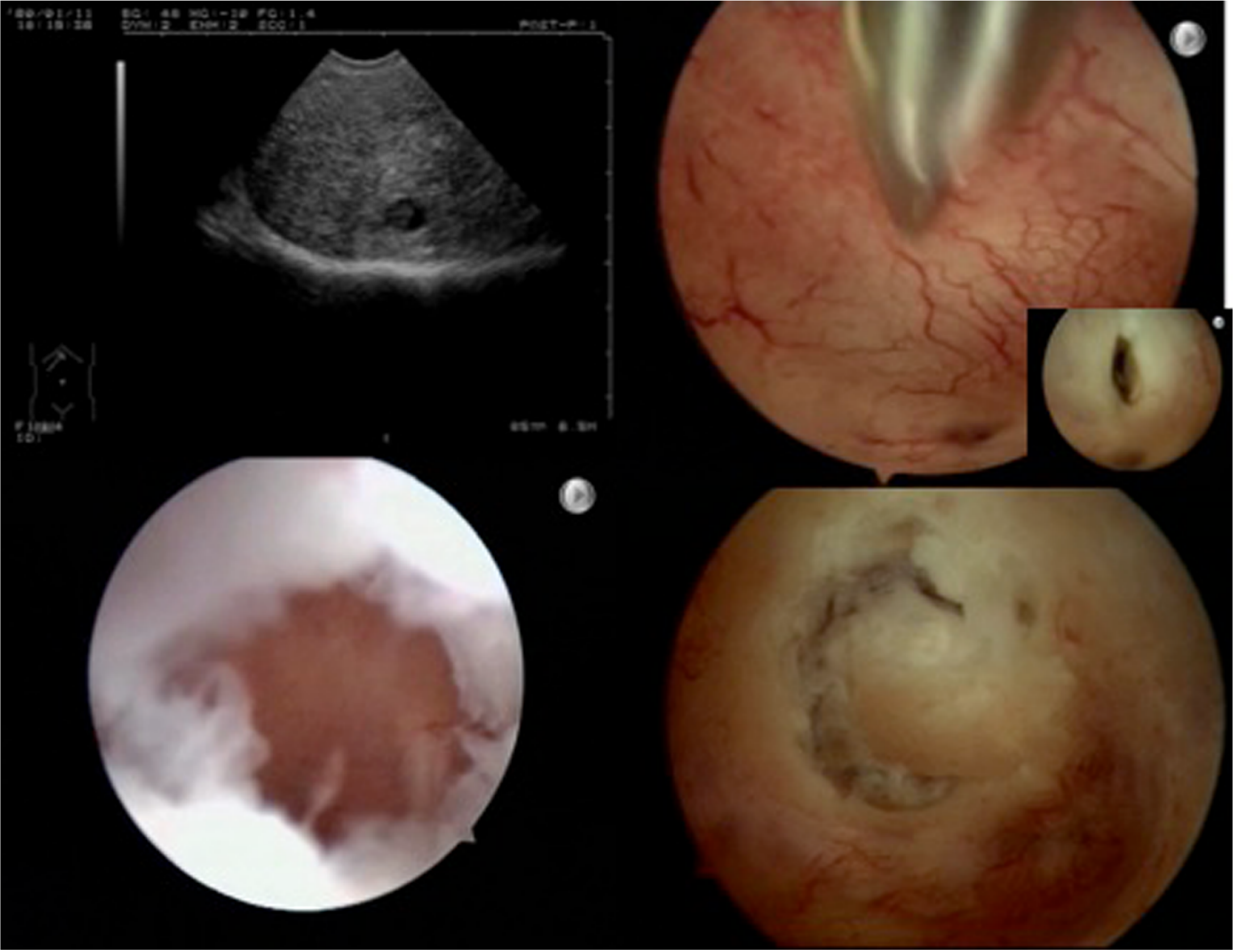
Case no. 1. **a** Cystic adenomyotic lesion at transvaginal ultrasound. **b** Abnormal vascularization and detail of the opened cyst after outflow of the brownish fluid. **c** Inside view of the cystic structure. **d** Dissection of cyst using 5-Fr scissors

Using a mini-hysteroscope and fluid as a distension medium, hysteroscopy was performed; it revealed the presence of a small opening in the fundus and a bulging area of abnormal vascularization on the anterior wall (Fig. 2). The abnormal vascularization became clearly visible after lowering the intrauterine pressure. Opening of the cystic bulging at the place of abnormal vascularization with 5-Fr scissors resulted in the outflow of a brownish fluid. An internal view of the cyst showed the presence of a somewhat fibrotic wall and areas of endometrial-like tissue. The lesion was completely resected with 5-Fr scissors.

**Fig. 3:**
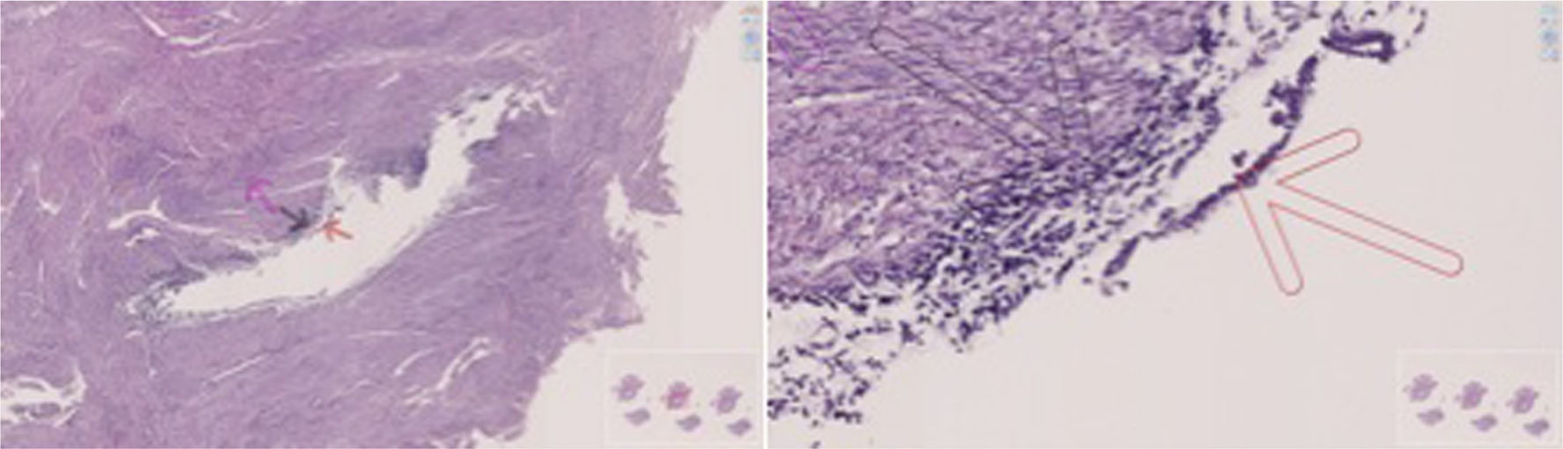
Histologic image. *Red arrow* endometrial epithelium. *Black arrow* stroma. *Purple arrow* myometrium

Histologic examination revealed a cystic structure lined by endometrial epithelium and surrounded by myometrium, compatible with benign cystic adenomyosis (Fig. 3).

### Case 2

A 38-year-old woman was referred to our center with a history of primary subfertility of 3-year duration. Previously, at another facility, a laparoscopic left salpingo-oophorectomy had been performed for severe endometriosis. An intramural cyst was diagnosed via MRI preoperatively, and the cyst was punctured and aspirated under ultrasound guidance during the operative laparoscopy. Because of recurrence of this intramural cyst, patient was referred to our center for hysteroscopic treatment.

**Fig. 4:**
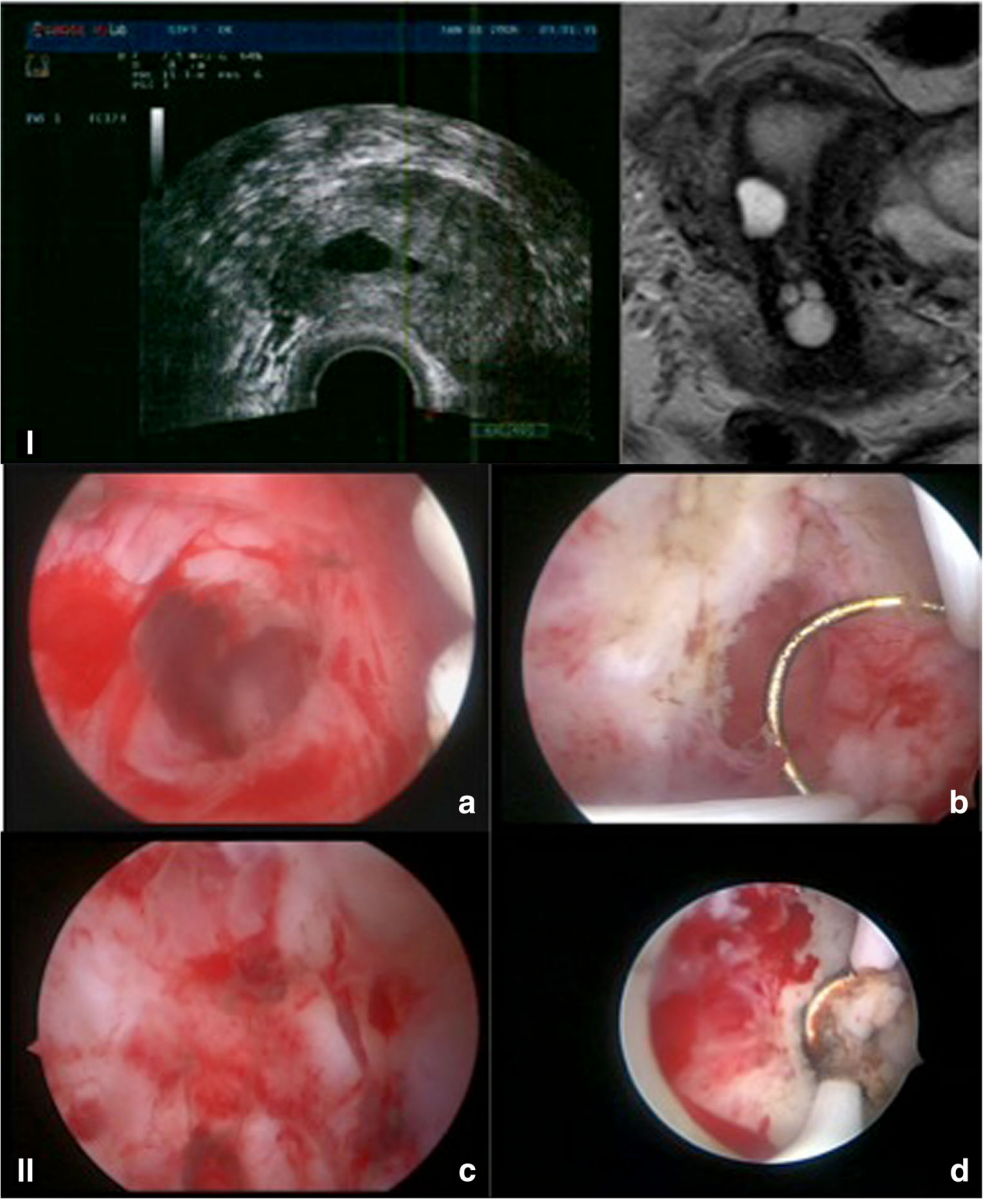
Case no. 2. **I** Ultrasound and MRI image of cystic lesion right isthmic part. **II** *a* Access to cystic structure after ultrasound-guided creation of channel to intramural cyst, *b* widening of access to cyst using a bipolar resectoscope, *c* insight view of cyst, *d* coagulation of insight cyst using a bipolar loop resectoscope

Vaginal ultrasound imaged the persistence of a cystic adenomyotic cyst at the isthmic level of the uterus (Fig. 4). At hysteroscopy, the endometrial cavity had a normal appearance and there were no direct visible signs of the presence of this cystic structure. Using the hysteroscope with ultrasound guidance, the cystic lesion was localized. After removal of the endoscope, the spirotome was inserted in the outer hysteroscopic sheet, and under ultrasound guidance, it was directed towards the cystic structure. A turning movement inserts the helix of the spirotome into the cystic structure. After withdrawal of the spirotome, the hysteroscope was reinstalled in the outer sheet. The spirotome created a clearly visible channel at hysteroscopic examination, allowing access to the cystic structure. Endometrial-like tissue was visible within the cyst. The inner cystic wall was coagulated using the small bipolar resectoscope and the bipolar coagulating probe. Another hysteroscopy 10 weeks after the first showed a normal uterine cavity with no adhesions and a slightly inflamed endometrial cavity. The patient underwent 2 months of GnRha therapy and was referred to our IVF program. Because a pregnancy did not develop following three IVF cycles and the patient suffered a recurrence of menorrhagia, another MRI was performed that revealed a focal enlargement of the junctional zone present at the mid-third of the uterine corpus. Another 3-month cycle of GnRha was planned before another IVF attempt.

## Discussion

The present cases illustrate the possibility of hysteroscopy for diagnosis and resection or ablation of intramural cystic adenomyosis. Although diagnostic hysteroscopy does not reveal the pathognomonic signs of adenomyosis, some studies suggests that an irregular endometrium with endometrial defects, altered vascularization, and cystic hemorrhagic lesion are possibly associated with adenomyosis [9].

With the use of MRI imaging and 3-D ultrasound adenomyosis, junctional zone hyperplasia and adenomyotic cysts can now be detected in younger patients during their reproductive years. The use of 3-D vaginal ultrasound for the diagnosis of a junctional zone abnormality or adenomyotic pathology has been extensively described by Exacoustos et al. [14] and can now be implemented in patients seeking fertility treatment. As patients are postponing their desire to conceive, we can expect an increasing incidence of adenomyotic pathology in patients with fertility problems.

Brosens et al. [2] suggested the acronym MUSCLE for the classification of the cystic adenomyosis: M *=* myometrial location (intramural, submucous, subserous), U *=* uterine site (midline, paramedian, lateral); S *=* structure (cystic, mixed, polypoid), C *=* contents (clear, hemorrhagic), L *=* level (fundus, body, cervix), and E *=* endometrial or inner lining (endometrium, metaplastic). The first patient can be classified as M(2)U(2)S(1)C(2)L(2)E(1) and the second as M(1)U(2)S(1)C(2)L(2)E(1). To distinguish the condition from other intramural cysts, histologic diagnosis is necessary to identify the endometrial inner layering and the presence of outer myometrium.

In cases of submucous cystic adenomyotic lesions or an adenomyoma bulging into the uterine cavity, direct hysteroscopic access is possible. Using 5-Fr scissors during hysteroscopy allows a clear dissection of the myometrial wall of the cyst from the surrounding myometrium. Instead of dissection with scissors, an ablative technique that destroys the inner cystic wall is a possible treatment option. We believe that this ablative approach is preferable for those cysts localized deeper in the intramural portion. In contrast to the healing process after hysteroscopic myomectomy, where a normal uterine cavity is expected, resection or ablation of adenomyotic cysts bulging into the uterine cavity results in a visible defect of the myometrium.

In cases in which intramural cystic structures are present, ultrasound guidance is mandatory for localization of the cystic structure. The spirotome offers the possibility of penetrating the cyst and leaving behind a visible channel allowing hysteroscopic entrance into the cystic structure for an ablative procedure using bipolar current.

It is currently unclear whether cases of intramural cystic adenomyosis, which do not communicate with the endometrial cavity, can be adequately treated with an ablative procedure or should be managed by enlarging the cyst opening to allow communication with the endometrial cavity.

The hysteroscopic technique has the advantage of leaving the outer myometrium intact and avoiding an abdominal scar. Because the incidence of adenomyosis is increasing with age and women are postponing their childbearing, increases of incidence of adenomyotic pathology in those patients referred for fertility treatment can be expected. Therefore, uterine exploration should not only focus on a careful inspection of the endometrial cavity but also entail an evaluation of the uterine wall with special attention to the junctional zone. This can routinely be performed in an office setting using 3-D vaginal ultrasound and office mini-hysteroscopy.

Cystic adenomyosis represents a specific entity of adenomyosis and has to be distinguished from other intramural cystic structures. The diagnosis necessitates a histologic examination showing endometrial epithelial lining of the inner cystic wall with surrounding outer myometrium. The differential diagnosis in adolescents includes the congenital anomaly of a hematometra in a non-communicating horn; therefore, hysteroscopy is indicated to observe both tubal openings and the presence of a normal endometrial cavity [15]. In younger women, Acien et al. [16] have described comparable cystic structures as accessory cavitated uterine masses (ACUMs). The lesions were primarily isolated cysts located at the insertion of the round ligament without infringement on the uterine cavity. He suggested that these lesions might arise from persistence of ductal Müllerian tissue near the round ligament. At present, there is no consensus regarding the most appropriate way to treat these lesions and the need of treatment for infertility patients. Thalluri and Tremelen [17] reported an impaired clinical pregnancy rate in patients with adenomyosis (23.6 versus 44.6 % in controls) undergoing IVF treatment using an antagonist ovarian stimulation protocol. A lower clinical pregnancy rate and higher spontaneous abortion rate are also reported in the meta-analysis of Vercellini et al. [18]. A conservative fertility-sparing technique is preferable. A laparoscopic cystectomy for a limited number of patients has been described in the Japanese literature [4, 5, 19, 20]. Takeuchi [4] described the use of laparoscopic cystectomy in nine cases with a reduction of pain postoperatively; two of three patients who desired to conceive became pregnant afterwards; however, the time between intervention and conception was 2 and 7 years. Also, Kriplani [21] reported on the laparoscopic treatment of four patients who experienced a reduction of pain postoperatively. Akar [22] described the use of robotics for performing a resection of cystic adenomyosis. Reduction of pain has been described after simple transvaginal aspiration of the fluid content [23].

If larger cystic adenomyotic structures localized in the outer intramural third are present, a laparoscopic approach is preferable. Although the cyst is unencapsulated (unlike the myoma), complete removal is possible, in contrast to focal or diffuse adenomyosis. Of the reported adenomyotic cysts [2], only 13 % had a diameter greater than 50 mm and 40 % were smaller than 25 mm. In these smaller structures, laparoscopic identification can be difficult. In these cases, laparoscopic access can cause significant trauma to the myometrial wall. Most laparoscopic resections have been performed for severe dysmenorrhea and/or dysfunctional uterine bleeding. Fertility was not a major factor in these patients. For patients desirous of pregnancy, a procedure with the highest fertility preservation and minimal trauma must be chosen. As in cases of a small adenomyoma, our experience showed that when smaller cystic structures were present in the inner third of the intramural part or submucosally, a minimally invasive hysteroscopic approach is possible. Some of the lesions are directly recognizable at hysteroscopy because they bulge into the endometrial cavity, thus favoring a minimally invasive dissection. For lesions localized deeper in the intramural portion, the spirotome can be introduced under ultrasound guidance; it creates a channel and provides hysteroscopic access to the cystic structure. Treatment by resection or ablation can then be performed.

Giana et al. [11] described the use of a bipolar resectoscope for the treatment of a cystic adenomyosis in a 46-year-old woman. In a report by Kumar [24], an intramural cyst was inadvertently opened during an ablative procedure for dysfunctional uterine bleeding. After exclusion of the possibility of uterine perforation, the procedure was continued. Histology confirmed the diagnosis of an adenomyotic cyst.

## Conclusions

The incidence of adenomyosis in patients with infertility is unclear. An estimated prevalence in patients with endometriosis has been reported to be approximately 70 % [25], and Brosens et al. [26] reported an incidence of 50 % in patients with subfertility, dysmenorrhea, and dysfunctional uterine bleeding. Cystic adenomyosis is a somewhat rare form of adenomyosis; however, with more women delaying pregnancy and with the availability of accurate and easy accessible indirect imaging techniques such as 3-D ultrasound, we can expect that more women seeking assisted reproduction will be diagnosed with adenomyosis and cystic adenomyosis. Together with office mini-hysteroscopy, a complete exploration of the uterus can now be performed including the endometrial cavity and the myometrial layers. Hysteroscopy offers the possibility of clear visualization of intracavitary lesions with a direct access to cystic adenomyosis. Treatment can be performed by mechanical dissection or bipolar ablative surgery. The spirotome allows performing a direct forward biopsy, and under ultrasound guidance, access is gained to intramural cystic lesions without visible intracavitary components. Hysteroscopy offers an alternative access for the treatment of cystic adenomyosis while producing minimal tissue damage. Further research is needed to better understand the impact of cystic adenomyosis on fertility and the possible beneficial effect of surgical removal.
